# Designing a Data Dashboard for Health Departments and Overdose Fatality Review Teams

**DOI:** 10.23889/ijpds.v9i5.2692

**Published:** 2024-09-18

**Authors:** Marie Pisani, Madeline Oguss, Claire Manneh, Anoop Mayampurath, Majid Afshar

**Affiliations:** 1 University of Wisconsin; 2 Datavant

## Introduction

Overdose Fatality Review (OFR) teams within health departments rely on data from multiple isolated sources to study the opioid crisis. Recently, we linked electronic health record data with state agency data to form a regional Substance Misuse Data Commons (SMDC) using privacy-preserving record linkage. Our goal in this study was to use human factors design principles to design a data dashboard for OFR teams using the linked datasets that will overcome current barriers within OFR workflows.

## Methods

We utilized NASA task load surveys, semi-structured interviews, and design sessions with end users to identify data needs for an optimal dashboard design. We assessed current workloads, data collection processes, and desired future state. We subsequently performed iterative design sessions for the generation and evaluation of low-fidelity prototypes. To overcome issues with privacy and security, we used synthetic data in a cloud-based platform to represent the SMDC for simulation.

## Results

Eleven OFR organizers participated. Pre-dashboard surveys on existing workflow showed high mental workload associated with data aggregation and case review, identifying a need for more accessible, comprehensive data. In our low-fidelity dashboard demo with synthetic data, iterative design adjustments were made in data visualization, storyline organizations, and theme-based data aggregation across pre-hospital and hospital data.

## Conclusions

We refined the data dashboard prototype into a high-fidelity version, set for further usability and human factors evaluation. We addressed privacy and security concerns through synthetic data use while the real-world data is maintained in a HIPAA-secure Azure cloud environment with access for approved users.

